# Evaluation of the Loop Mediated Isothermal DNA Amplification (LAMP) Kit for Malaria Diagnosis in *P. vivax* Endemic Settings of Colombia

**DOI:** 10.1371/journal.pntd.0003453

**Published:** 2015-01-08

**Authors:** Andrés F. Vallejo, Nora L. Martínez, Iveth J. González, Myriam Arévalo-Herrera, Sócrates Herrera

**Affiliations:** 1 Caucaseco Scientific Research Center, Cali, Colombia; 2 Foundation for Innovative New Diagnostics (FIND), Geneva, Switzerland; 3 Faculty of Health, Universidad del Valle, Cali, Colombia; Federal University of São Paulo, Brazil

## Abstract

**Background:**

Most commonly used malaria diagnostic tests, including microscopy and antigen-detecting rapid tests, cannot reliably detect low-density infections which are frequent in low transmission settings. Molecular methods such as polymerase chain reaction (PCR) are highly sensitive but remain too laborious for field deployment. In this study, the applicability of a malaria diagnosis kit based on loop-mediated isothermal amplification (mLAMP) was assessed in malaria endemic areas of Colombia with *Plasmodium vivax* predominance.

**Methodology/Principal Findings:**

First, a passive case detection (PCD) study on 278 febrile patients recruited in Tierralta (department of Cordoba) was conducted to assess the diagnostic performance of the mLAMP method. Second, an active case detection (ACD) study on 980 volunteers was conducted in 10 sentinel sites with different epidemiological profiles. Whole blood samples were processed for microscopic and mLAMP diagnosis. Additionally RT-PCR and nested RT-PCR were used as reference tests. In the PCD study, *P. falciparum* accounted for 23.9% and *P. vivax* for 76.1% of the infections and no cases of mixed-infections were identified. Microscopy sensitivity for *P. falciparum* and *P. vivax* were 100% and 86.1%, respectively. mLAMP sensitivity for *P. falciparum* and *P. vivax* was 100% and 91.4%, respectively. In the ACD study, mLAMP detected 65 times more cases than microscopy. A high proportion (98.0%) of the infections detected by mLAMP was from volunteers without symptoms.

**Conclusions/Significance:**

mLAMP sensitivity and specificity were comparable to RT-PCR. LAMP was significantly superior to microscopy and in *P. vivax* low-endemicity settings and under minimum infrastructure conditions, it displayed sensitivity and specificity similar to that of single-well RT-PCR for detection of both *P. falciparum* and *P. vivax* infections. Here, the dramatically increased detection of asymptomatic malaria infections by mLAMP demonstrates the usefulness of this new tool for diagnosis, surveillance, and screening in elimination strategies.

## Author Summary

The ability to detect and treat asymptomatic infections will be fundamental to eliminate malaria. This requires highly sensitive screening tests close enough to cases to enable rapid treatment. Very low parasitemias can be detected by molecular methods such as PCR; however, these techniques require considerable training and are restricted to reference laboratories. A new field-stable diagnostic kit for malaria based on loop-mediated isothermal DNA amplification (LAMP) is now commercially available. This LAMP kit is able to detect down to 1 parasite/µl of blood in less than 1 hour. In order to evaluate the feasibility of this LAMP kit as a tool for the detection of asymptomatic malaria in malaria endemic areas of Colombia with *Plasmodium vivax* predominance, we conducted field studies using the LAMP kit implemented in remote settings. We found that LAMP sensitivity and specificity were comparable to RT-PCR for detection of both *P. falciparum* and *P. vivax* infections and dramatically increased detection of asymptomatic malaria infections. This simple detection method for very low parasitemia raises opportunities and new strategies for malaria elimination.

## Introduction

Malaria causes over 207 million clinical cases and ∼627,000 deaths worldwide every year representing an enormous global social and economic burden [1]. However, the distribution is not equal: the African continent is a highly-endemic region accounting for ∼80% of the worldwide malaria cases compared to regions like the American continent which contributes only a minor percentage of the global malaria with ∼469,000 cases reported in 2012 [1]. During the last decade, several countries of this region including Argentina, Belize, Bolivia, Costa Rica, Ecuador, El Salvador, French Guiana, Guatemala, Honduras, Mexico, Nicaragua, Paraguay and Suriname have experienced a drastic decrease in malaria case incidence (>75% reduction) and some of these countries are currently in the pre-elimination phase [1], [2].

Although microscopic detection of *Plasmodium* species on thick blood smear (TBS) has been the standard method for malaria diagnosis for decades and currently represents the gold standard diagnostic method for malaria control programs, its field accuracy, errors in species identification and its operator-dependence significantly limits its usefulness particularly in regions of low malaria transmission with high prevalence of asymptomatic low-density infections. Microscopy has usually been considered less expensive and easier to handle than other malaria diagnostic methods developed more recently such as nucleic acid amplification test (NAAT); however, it is laborious, time consuming, and requires well trained personnel which are frequently not available in remote malaria-endemic areas. This creates uncertainty about the cost-effectiveness of microscopy in low transmission settings [3].In this context, new diagnostic methods based on detection of parasite antigens by monoclonal antibodies which are currently grouped under the name of rapid diagnostic tests (RDTs) have shown great performance in terms of sensitivity and specificity. RDTs represent a tool which is highly complementary to microscopy. Their simplicity has made them attractive to provide diagnosis at all levels of the health care systems, particularly in remote areas which have limited service infrastructure and where health workers have limited training and supervision. However, RDTs still present limitations such as batch-to-batch variation and limited sensitivity at low parasitaemia (<200 parasites/µL). Novel techniques based on parasite nucleic acid detection, particularly those based on the use of polymerase chain reactions (PCR), have recently proven to display greater sensitivity and specificity compared to microscopy and RDTs [3]–[5]. These two features are highly desirable in areas of low malaria transmission intensity where a great proportion of asymptomatic cases are submicroscopic. These asymptomatic cases remain as reservoirs for malaria transmission, limiting the progress of malaria elimination programs [3], [6]. However, until now, PCR based techniques required expensive and complex infrastructure as well as significant training. The loop-mediated isothermal amplification of DNA (LAMP) is a molecular method that also detects specific genes in the genome of a target microorganism. LAMP is similar to conventional PCR in that both techniques amplify DNA. However, in contrast to PCR, LAMP is performed with less sophisticated equipment such as a water-bath and an ultraviolet-lamp and does not require a thermocycler for amplification nor a gel imaging system for result reading [7]. The LAMP technique is highly efficient for DNA amplification (up to 10^10^ times amplification within 15 to 60 minutes), it is faster than PCR, and it can be performed in basic laboratories without the need for specialized infrastructure.

Using nested PCR as reference standard [8], a new LAMP kit amplifying *Plasmodium* mitochondrial DNA (mLAMP) displayed sensitivities ranging between 89.5–93.3% and a specificity between 95.9–100% [7], [9]. In those studies, a simple 15 minutes boil and spin DNA extraction method was used without affecting the performance of the mLAMP kit.

Diagnostic tools like mLAMP which are capable of detecting very low parasite densities (1 parasite/µL) and reliable under field-conditions are urgently needed for active case detection to accelerate malaria elimination [10]. The mLAMP kit currently available in the market detects all *Plasmodium* species and allows for *P. falciparum* speciation. New versions of this kit are in development [11] which will allow for *P. vivax* speciation [7] and higher throughput for population screening. Here we assessed the performance and the capability of the available mLAMP kit to detect submicroscopic infections in malaria endemic settings of Colombia with different transmission intensities and where both *P. vivax* and *P. falciparum* are present at different proportions.

## Materials and Methods

A total of 10 sentinel sites were selected from three well characterized malaria endemic setting of Colombia with different malaria transmission intensities and different prevalence of *P. vivax* and *P. falciparum* malaria parasites:

Tierralta is a municipality located in the Department of Cordoba in the northwestern part of Colombia with ∼90,000 inhabitants composed of ∼2% indigenous-groups and 44.4% rural population. The predominant malaria parasite species in this region is *P. vivax* (82.4%) followed by *P. falciparum* (17.4%) and mixed malaria infections (0.2%) [12]. The selected sentinel sites in this region were: Los Pollos, El Loro, Tuis Tuis and La Unión.

Buenaventura is located on the Pacific coast of Valle Department in the west of Colombia, with ∼350,000 inhabitants which are predominantly Afro-Descendant (72.4%). The predominant malaria parasite species in the region is *P. vivax* (85%) followed by *P. falciparum* (15%). The sentinel sites selected in this region were Punta Soldado, Zacarias and La Delfina.

Tumaco is located in the Department of Nariño in the southwest of the country near the Ecuador border. It has a population of ∼187,084 inhabitants composed mainly of Afro-Descendants (88%). The predominant malaria parasite species in the region is *P. falciparum* (79.2%) followed by *P. vivax* (20.8%). The sentinel sites selected in this region were Robles, Candelillas and Bucheli.

### Study Subjects

#### Passive case detection (PCD)

278 febrile adults and children attending San Jose Hospital in Tierralta between April and December, 2013 were invited to participate in the study and requested to sign an informed consent (IC) previously approved by the Caucaseco Scientific Research Center Institutional Review Board (IRB). For mLAMP processing, whole blood were collected from each patient by arm venipuncture using heparinized tubes. TBS was done from finger prick and PCR from EDTA tubes.

#### Active case detection (ACD)

A total of 980 adult and children volunteers were enrolled under a cross-sectional survey carried out in the ten sentinel sites between October and December 2013 during the rainy season despite the transmission in Colombia is perennial. The cross- sectional study was performed in two steps: a) a door-to-door census of the entire population of each site and b) blood-sample collection in volunteers from a subset of randomly selected houses. After written IC/assents were obtained from adult and minors (1–17 years of age) and their parents/guardians, respectively, a trained member of the study staff collected clinical, demographic and epidemiological information from each participant using a standardized questionnaire. Additionally, a trained team of physicians collected the blood for lab tests (1 mL/each) which were processed as described below. Microscopy and mLAMP were performed in the field whereas RT-PCR was performed in Caucaseco Scientific Research Center (SRC) in Cali, Colombia. For mLAMP processing, blood samples (60 µL) were collected from finger pricks using disposable Pasteur pipets and delivered into vials containing 60 µL of lysis buffer (0.4% SDS, 400 mM NaCl, 40 mM Tris pH 6.5) that were maintained at room temperature (25–30°C) for ∼24 hour until reach the processing site.

### Laboratory Analyses

For training and initial validation of mLAMP, a subset of samples with confirmed malaria diagnosis was used. A total of 150 blood samples were extracted by the boil and spin method and were evaluated using the mLAMP kit. The results were compared to those of RT-PCR performed using purified DNA (Invitrogen, Purelink DNA). Discordant samples were tested in triplicates using nested RT-PCR (3-well RT-PCR).

#### Microscopic diagnosis

Using approximately 100 µL of blood previously collected by finger prick, both thick and thin duplicate smears were prepared, stained with Giemsa stain [13] and examined under oil immersion by an expert microscopist with over 10 years of experience. Parasitaemia was confirmed by a second experienced reader. For parasite density, at least 500 leucocytes were reviewed before scoring a sample as negative. Total parasite load was expressed as the number of parasites/µL and assuming a leukocyte count of 8,000/µL.

#### Training on LAMP procedures

Two laboratory technicians in each sentinel site and with no previous experience on molecular methods were trained during 3 days on LAMP procedures for samples collection, processing, amplification and detection. Demonstration of procedures were done during day 1, supervised performance of the test during day 2, and blind testing of reference samples during day 3.

#### DNA extraction for mLAMP

In Tierralta and Buenaventura, DNA extraction and mLAMP were performed in small local laboratories whereas samples in lysis buffer from Tumaco were sent by plane for processing in Cali. Stability of blood in lysis buffer at 37°C for 24 h was assessed to ensure quality of DNA (S1 Text). DNA extraction was performed by the boil and spin method as previously described [7]. Briefly, 60 µL of blood in 60 µL of extraction buffer (0.4% SDS, 400 mM NaCl, 40 mM Tris pH 6.5) were heated to 95°C for 5 minutes. After centrifugation at 10,000 g for 3 minutes, 25 µL of clear supernatant were diluted with 287 µL of distilled water and 25 µL per reaction tube used for mLAMP amplification (equivalent to 1 µL of whole blood).

#### Malaria LAMP amplification reaction

mLAMP kits (Loopamp MALARIA Pan/Pf detection kit - LMC 562, Eiken Chemical Co., Ltd. Tokyo, Japan) were transported and stored at ambient temperature and used before the expiration date as recommended by the manufacturer. For each sample, 25 µL of diluted DNA were transferred into reaction tubes with dried reagents designed to detect all *Plasmodium* species (Pan-mLAMP) as well as specifically detecting *P. falciparum* (*Pf*-mLAMP). All reaction tubes were placed into a LAMP incubator for DNA amplification at 65°C for 40 minutes and reaction inactivation at 80°C for 5 minutes. Results were read by visual fluorescence by two independent readers and a third reader was called if discordances occurred. Positive and negative controls were included for each batch of reactions. For mLAMP implementation in the field, laboratory personnel and field technicians were trained on all study procedures in a three-day workshop. Complete standard operating procedures for the mLAMP assay and boil and spin method are available online [14].

#### Real-time PCR (RT-PCR)

Assays were performed as described previously [15] with minor modifications. DNA was extracted using the PureLink Genomic DNA kit. The amount of DNA in each well was adjusted to be equivalent to 1 µL of whole blood. Standard *P. falciparum* and *P. vivax* DNA positive and negative controls were used in each batch of tests including the extraction of both negative and inhibition control. A sample was considered negative if there was no increase in the fluorescent signal after a minimum of 40 cycles. Parasitemia quantification was performed using a parasite specific standard curve made with serial blood dilutions of a reference field isolate. Each reaction plate included a standard curve for parasite quantification.

#### Malaria nested RT-PCR

For positive samples, DNA was extracted using the PureLink genomic DNA kit and 2 µL of DNA were used to carry out the species-specific nested RT-PCR [8]. In the first amplification reaction, a pair of genus-specific primers was used to amplify a 1.6 Kb fragment of the DNA coding sequence of the ribosomal 18S subunit of any *Plasmodium* species. The second PCR reaction was developed as previously described [16] using a 10 fold dilution of the PCR product from the first reaction.

#### Testing discordant samples

LAMP results with Pan and *Pf* assays were analyzed independently. All mLAMP and RT-PCR discordant samples and an equal number of randomly selected concordant samples (both for Pan and *Pf* LAMP assays) were re-tested in a blinded fashion by mLAMP and nested RT- PCR in triplicates. The sample was declared as positive if at least 1/3 reaction well was positive.

### Data Analysis

#### Data entry

Information was captured in the field in paper-based case report forms (CRF). Data was digitized using REDCap (version 4.1) and imported into MATLAB (version 2011b) for analysis.

#### Data quality assurance

The data quality assurance (QA) procedure consisted of setting a quality control (QC) sample of size q less than the total number p of all CRFs for each location. The maximum permissible error for this study was set to 1%.

#### Statistical analysis

Data analysis was performed using MATLAB (version 2011b). Using descriptive statistics, the general characteristics of individuals admitted to the study were established. Measures of central tendency and dispersion were calculated for qualitative characteristics, whereas absolute frequencies as well as confidence intervals were used for quantitative characteristics. The primary analysis was conducted using RT-PCR as gold standard to determine diagnostic performance characteristics (sensitivity, specificity, negative (NPV) and positive (PPV) predictive values, and overall diagnostic accuracy) of mLAMP.

### Ethics Statement

This study was carried out in accordance with institutional guidelines. The protocol was previously reviewed and approved by the IRB of Caucaseco Scientific Research Center (CECIV, Cali-Colombia). Written informed consent (IC) was obtained from each volunteer at enrollment. Parents were asked for consent for their child to take part in the study. Information obtained from the participants was managed on principles of confidentiality. Immediately after blood donation, malaria positive volunteers were provided with the antimalarial treatment recommended by the Colombian Ministry of Health.

## Results

### Initial Validation of mLAMP in a Reference Laboratory

An initial assessment of the mLAMP kit was done with 150 well characterized blood samples (70 *P. vivax*, 30 *P. falciparum* and 50 *Plasmodium* negative samples) at the Caucaseco SRC. The sensitivity and specify of mLAMP using both Pan and Pf reaction tubes under laboratory conditions were 81% (95%CI: 75–93) and 100% (95%CI: 87–100) respectively for *P. falciparum* whereas for *P. vivax* the sensitivity and specificity were 98% (95%CI: 89–100) and 82% (95%CI: 73–89) respectively. Most of the false negative results (3 out of 4) corresponded to samples with low density *P. vivax* infections as determined by RT-PCR.

### mLAMP Performance for PCD

A total of 278 febrile subjects were tested for malaria in Tierralta using mLAMP (S1 Fig.). The main demographic features of the cohort are described in Table 1. The proportion of female participants was 60.8% and the population was predominantly between 11 and 30 years of age (50.4%). None adverse event was observed. The prevalence of microscopically confirmed malaria cases among the recruited patients was 28.1%, 10 *P. falciparum* cases (120–6,500 parasites/µL) and 70 *P. vivax* cases (320–16,000 parasites/µL). All the case were non-complicated malaria. 94 out of the total 278 samples prepared by the boil and spin method (33.8%) were positive with Pan-mLAMP malaria specific primers. To determine Pan-mLAMP performance, a corrected data base was obtained after adjusting the discordant results using nested RT-PCR as reference. Pan-mLAMP displayed 100%, sensitivity, 98.9% specificity, 76.9% PPV and 100% NPV for *P. falciparum*. In contrast, for *P. vivax* samples, Pan-mLAMP displayed 91.4%, sensitivity, 91.8% specificity, 80.5%, PPV, and 99.6% NPV (Table 2).

**Table 1:** Demographics of study population in Tierralta, Buenaventura, and Tumaco.

	Passive case detection		Active case detection	
	N	%	n	%
No participants	278	100	980	100
Male sex	168	60.4	398	40.6
Age 0–4	3	1.1	65	6.6
Age 5–17	63	22.6	345	35.2
Age >18	212	76.3	567	57.9

Colombia. April to December 2013.

**Table 2:** Diagnostic performance of microscopy and mLAMP kit (Pan and *Pf* reaction tubes) for passive case detection (PCD) and compared to 3-well nested RT-PCR as reference standard (n = 278).

Method and Parasite species	Parameter	%	95% CI
Microscopy	Sensitivity	86.1	66.0 to 83.5
*P. vivax*	Specificity	97.6	97.8 to 99.0
	PPV	74.8	65.2 to 82.8
	NPV	98.5	97.8 to 99.0
Microscopy	Sensitivity	100	69.2 to 100
*P. falciparum*	Specificity	99.3	97.3 to 99.9
	PPV	38.6	51.6 to 97.9
	NPV	99.3	98.6 to 100
Pan-mLAMP	Sensitivity	91.4	82.3 to 96.8
*P. vivax*	Specificity	91.8	87.2 to 95.1
	PPV	80.5	68.5 to 87.2
	NPV	99.6	93.5 to 98.9
Pan-mLAMP	Sensitivity	100	69.2 to 100
*P. falciparum*	Specificity	98.9	96.7 to 99.7
	PPV	76.9	44.8 to 95.4
	NPV	100	98.6 to 100
Pf-mLAMP	Sensitivity	100	69.2% to 100
*P. falciparum*	Specificity	99.3	97.3% to 99.9
	PPV	83.3	51.6% to 97.9
	NPV	100	98.6% to 100
Microscopy	Sensitivity	86.1	66.0 to 83.5
*P. vivax*	Specificity	97.6	97.8 to 99.0
	PPV	74.8	65.2 to 82.8
	NPV	98.5	97.8 to 99.0
Microscopy	Sensitivity	100	69.2 to 100
*P. falciparum*	Specificity	99.3	97.3 to 99.9
	PPV	38.6	51.6 to 97.9
	NPV	99.3	98.6 to 100
Pan-mLAMP	Sensitivity	91.4	82.3 to 96.8
*P. vivax*	Specificity	91.8	87.2 to 95.1
	PPV	80.5	68.5 to 87.2
	NPV	99.6	93.5 to 98.9
Pan-mLAMP	Sensitivity	100	69.2 to 100
*P. falciparum*	Specificity	98.9	96.7 to 99.7
	PPV	76.9	44.8 to 95.4
	NPV	100	98.6 to 100
Pf-mLAMP	Sensitivity	100	69.2% to 100
*P. falciparum*	Specificity	99.3	97.3% to 99.9
	PPV	83.3	51.6% to 97.9
	NPV	100	98.6% to 100

### Pan-mLAMP Performance for ACD

Main demographic features of the enrolled volunteers in the 10 sentinel sites are described in Table 1. In this cross-sectional surveys of 980 volunteers, a total of 71 positive infections were identified by RT-PCR (1–897 parasites/µL), with 22.5% (n = 16) *P. falciparum*, 77.5% (n = 55) *P. vivax* and 0% mixed infections. Pan-mLAMP detected 98.0% of these asymptomatic infections confirmed by RT-PCR, most of which occurred among participants >15 years old. Malaria prevalence across all sentinel sites was 0.2% by microscopy (n = 2) and 6.6% by Pan-mLAMP (n = 65). Comparison of Pan-mLAMP with corrected single-well RT-PCR for *P. falciparum* indicated that Pan-mLAMP had 100% sensitivity, 99.9% specificity, 93.8% PPV and 100% NPV. In the case of *P. vivax* samples, Pan-mLAMP presented 90.9% sensitivity, 99.5% specificity, 90.9% PPV and 99.5% NPV (Table 3). A total of 11 samples remained discordant: one false negative by Pan-mLAMP for *P. falciparum*, five false positive by Pan-mLAMP and five false negative for *P. vivax* (S2 Fig.).

**Table 3:** Diagnostic performance of microscopy and Pan-mLAMP kit for active case detection (ACD) of asymptomatic infections and compared to 3-well nested RT-PCR as reference standard (n = 980).

Method and Parasite species	Parameter	%	95% CI
Microscopy	Sensitivity	0	0 to 6.5
*P. vivax*	Specificity	100	99.6 to 100
	PPV	—	
	NPV	9.4	92.80 to 95.7
Microscopy	Sensitivity	5.9	0.2 to 28.7
*P. falciparum*	Specificity	99.9	99.4 to 100
	PPV	50.0	0.04 to 99.9
	NPV	98.4	97.36 to 99.0
Pan-mLAMP	Sensitivity	90.9	80.0 to 97.0
*P. vivax*	Specificity	99.5	98.7 to 99.8
	PPV	90.9	79.9 to 97.0
	NPV	99.5	98.7 to 99.8
Pan-mLAMP	Sensitivity	100	78.2 to 100
	Specificity	99.9	99.4 to 100
*P. falciparum*	PPV	93.8	69.8 to 99.8
	NPV	100	99.6 to 100

## Discussion

The malaria LAMP kit used in this study and under minimum infrastructure conditions achieved sensitivity and specificity comparable to RT-PCR performed in a reference laboratory. Similar results have been reported in previous studies [7]. One previous study demonstrated reliable detection of parasites at a very low threshold in a remote clinical setting under passive case detection [9] and in others studies, malaria LAMP assays was used for detection of parasite from *in vitro* cultures or field samples transported to research laboratories [17]–[19]. To our knowledge, the current study is the first to demonstrate high sensitivity and specificity of mLAMP kit for both *P. falciparum* and *P. vivax* infections and the reliable detection of asymptomatic infections under active case detection in remote settings. Additionally, this study demonstrates that technicians who had no previous training in molecular diagnostic techniques and just 3 days of training in LAMP procedures were able to perform the test in a large number of samples under field-conditions.

When using mLAMP for PCD, a higher proportion of positive patients was detected (33.1%) when compared to microscopy (28.1%). For *P. falciparum*, similar sensitivity and specificity were observed between RT-PCR and mLAMP. For *P. vivax*, all the discordant results were resolved in favor of mLAMP. Therefore, under PCD of the cohort of febrile patients, mLAMP detected 5% more malaria cases.

A significant number of infections with low parasite density were detected when using mLAMP for ACD. The kit achieved a sensitivity equivalent to RT-PCR in the detection of infections of low malaria parasite density. The estimate of malaria prevalence by mLAMP was 6.1% (n = 60) with mostly asymptomatic cases (59/60). The identification and management of asymptomatic carriers has become a new and increasingly important challenge for malaria control programs [3], [20]. The treatment of asymptomatic carriers as part of routine surveillance strategies has the potential to make a significant contribution to the reduction of malaria transmission in endemic regions [21]. Undetected and untreated asymptomatic infections may result in a continuous source of gametocytes for local mosquito vectors [22]. Whereas clinically gametocytes are considered to be harmless, in terms of public health, gametocytes play a central role in the maintenance of malaria transmission and therefore should be detected and treated promptly.

The processing time for mLAMP in each of the sentinel sites was <1 day, whereas PCR results were available in 7 days. This significant time reduction would directly translate into timely initiation of treatment and reduction of patient withdrawal from treatment. mLAMP requires less training, is faster and requires minimal equipment compared to RT-PCR. The combination of high sensitivity and specificity together with the ease and efficiency of performance and stability at ambient temperature makes mLAMP a promising new tool for malaria surveillance, particularly for the detection and prompt treatment of asymptomatic infections in low transmission settings. In regions of declining malaria transmission, the use of highly sensitive tools such as mLAMP in remote clinics would contribute to the reduction of transmission and to the acceleration of malaria elimination.

The current format of the Pan-*Plasmodium* genus and *P. falciparum*–specific LAMP kit is able to identify all *P. falciparum*–infected individuals. Non-falciparum *Plasmodiu*m species are also detected but not resolved to the species level, a limitation shared with most RDT tests. Moreover, under the current configuration, the kit does not differentiate mixed infections from *P. falciparum* mono infections. Despite this limitation, by the combined use of the Pan-malaria and *P. falciparum* kits, we could identify all *P. vivax* cases as confirmed by RT-PCR. Reagents able to identify infections with other species and more specifically with *P. vivax* parasites, as well as mixed infections, are required to guide appropriate treatment of malaria cases in the clinical setting.

### Conclusions

This malaria mLAMP kit is an easy-to-use and field stable diagnostic tool which brings molecular techniques to areas with low laboratory infrastructure. The availability of this technique opens news perspectives in the implementation of surveillance and response activities in malaria elimination campaigns. The detection and prompt treatment of sub-microscopic asymptomatic infections in less than one hour after sample collections may contribute to the control of transmission and accelerate malaria elimination.

## Supporting Information

S1 Table **STARD checklist.**
(DOCX)

S1 Fig **STARD flow chart for PCD S2.**
(TIF)

S2 Fig **STARD flow chart for ACD S3.**
(TIF)

S1 Text **Sample stability.**
(DOCX)
