# Supplementary figures and images for: Evaluation of the Loop Mediated Isothermal DNA Amplification (LAMP) Kit for Malaria Diagnosis in P. vivax Endemic Settings of Colombia
Source: PLoS Negl Trop Dis. 2015 Jan 8;9(1):e3453. doi: 10.1371/journal.pntd.0003453 (PMC4287555; doi:10.1371/journal.pntd.0003453)

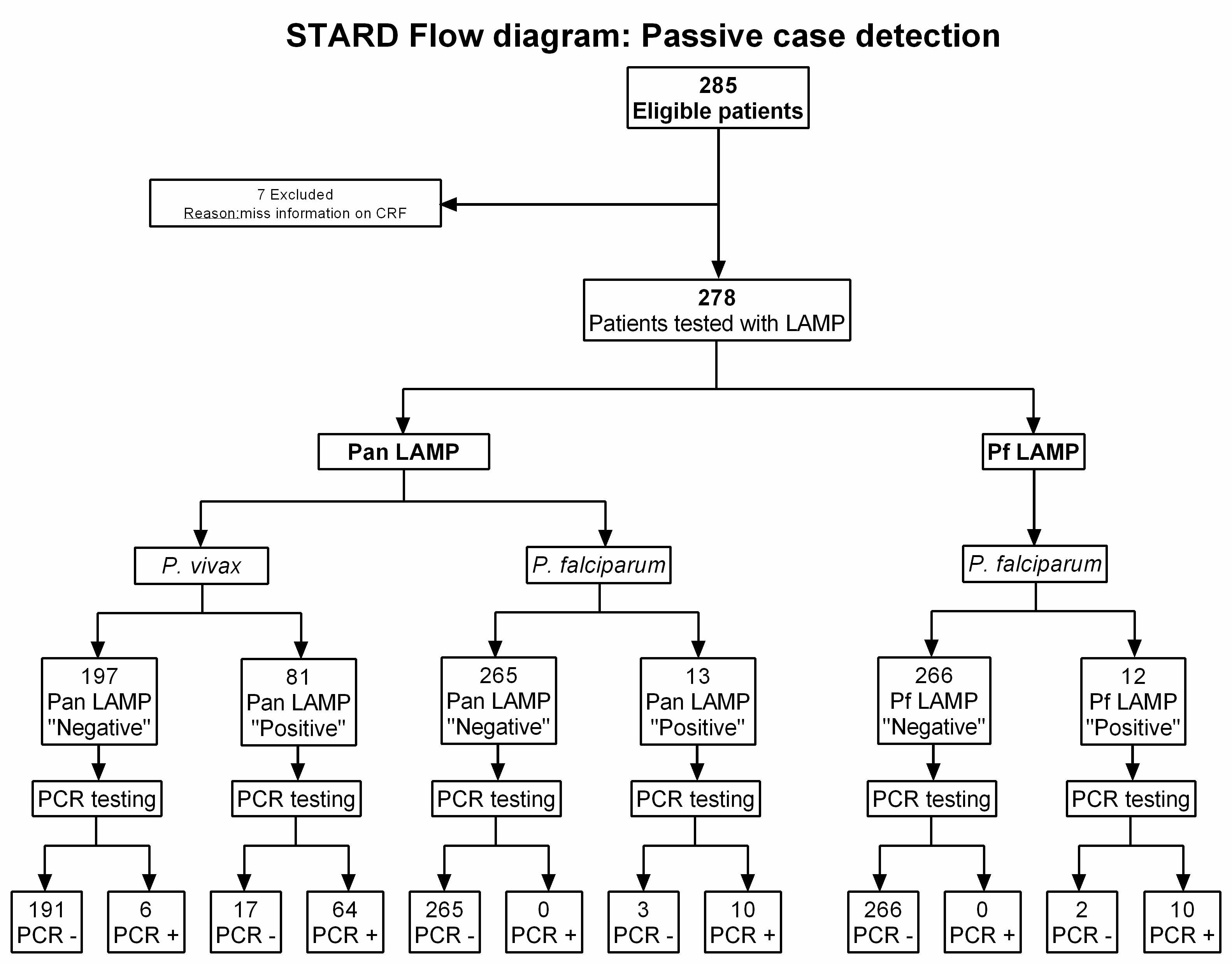

Supplement: S1 Fig — STARD flow chart for PCD S2. (TIF) [file pntd.0003453.s002.tif]

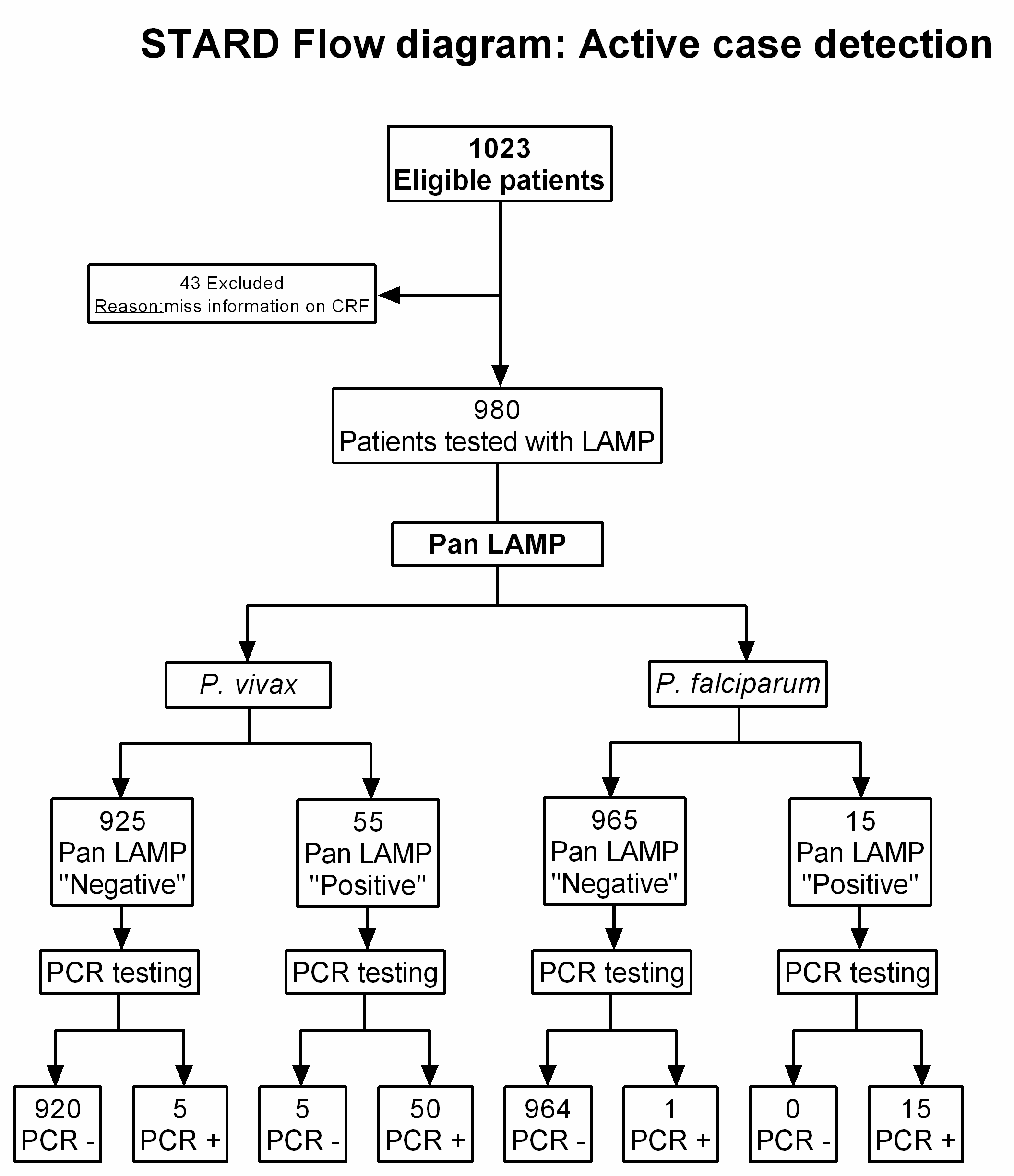

Supplement: S2 Fig — STARD flow chart for ACD S3. (TIF) [file pntd.0003453.s003.tif]
