# Supplementary material for: Evaluation of the Loop Mediated Isothermal DNA Amplification (LAMP) Kit for Malaria Diagnosis in P. vivax Endemic Settings of Colombia
Source: PLoS Negl Trop Dis. 2015 Jan 8;9(1):e3453. doi: 10.1371/journal.pntd.0003453 (PMC4287555; doi:10.1371/journal.pntd.0003453)
Supplement: S1 Text — Sample stability. (DOCX) [file pntd.0003453.s004.docx]

**Sample stability for field studies**

**Methods**

Two recent blood samples from patients infected with *P. falciparum* were selected for this study from blood stored at 4 degrees. The first sample was less than 24 hours old and contained approximately 150,000 parasites per μl as determined by expert microscopy. This sample was diluted down in uninfected EDTA blood which had been washed in 0.9% NaCl to remove the anticoagulant. The sample was diluted down to 7 and 3.5 parasites per μl for use. The second sample was a three days old EDTA venipuncture containing approximately 5 parasites per μl as determined by expert microscopy. This sample was not processed in any way before use.

60 μl of each blood was aliquoted into a 0.5ml snap top tube. For samples A and C 60ul of rapid boil lysis buffer was also added to the tube and then the samples mixed by pulse vortexing. Tubes were stored at either 37 deg C or 4 deg C (Table 1). Samples were then subjected to rapid boil and spin according to the SOP devised by FIND. 2 μl of the supernatant was removed and put into 28 μl of ddH2O in either a Pg or Pf tube and the tubes then reconstituted and run in an LA-320CE turbidimeter according the FIND’s SOP.

| Code | Contents | | Storage for 24 hours |
| --- | --- | --- | --- |
| A | blood + buffer | | 37 degrees |
| B | blood only |  | 37 degrees |
| C | blood + buffer | | 4 degrees |
| D | blood only |  | 4 degrees |

Table 1

**Results**

All samples were positive using Pan tubes in blood samples from all four different storage conditions. We have previously proven that very little degradation of the DNA occurs when whole blood is stored at 4 degrees C. Some apparent retardation of the time to positive turbidimetry is apparent in the samples stored at 37 degrees (both in buffer and out of buffer) compared to those stored at 4 degrees. However all samples were still positive even at 3 parasites per μl (Figure 1).

Figure 1. LAMP amplification in samples stored at 37 and 4 degrees.

**Conclusions**

When used neat, storage at any temperature in lysis buffer does not seem to adversely affect the stability of amplifiable DNA in malaria infected bloods. This is true with samples containing only 3 parasites per μl, exceptionally near the intended LOD of the kit.
